# Acromegaly diagnosis

**DOI:** 10.1210/clinem/dgag189

**Published:** 2026-04-29

**Authors:** Divya Yogi-Morren, Philippe Chanson

**Affiliations:** Department of Endocrinology, Diabetes & Metabolism, Medical Specialty Institute, Cleveland Clinic, Cleveland, OH 44195, USA; Université Paris-Saclay, Inserm, Physiologie et Physiopathologie Endocriniennes, Assistance Publique-Hôpitaux de Paris, Hôpital Bicêtre, Service d’Endocrinologie et des Maladies de la Reproduction, Centre de Référence des Maladies Rares de l’Hypophyse, Le Kremlin-Bicêtre 94275, France

**Keywords:** acromegaly, growth hormone, insulin-Like growth factor 1, diagnosis, artificial intelligence, comorbidity clusters

## Abstract

The clinical presentation of acromegaly reflects systemic effects of chronic growth hormone (GH) and insulin-like growth factor 1 (IGF-I) excess. Diagnostic delay frequently ranges from 6 to 10 years. While classical manifestations such as acral enlargement and facial coarsening are diagnostically important, many patients initially develop nonspecific symptoms, including sleep apnea, carpal tunnel syndrome, arthralgia, and metabolic disturbances. The lack of symptom/comorbidity specificity highlights the need for improved screening strategies, particularly for patients without overt acral changes. Comorbidity cluster analyses, potentially supported by artificial intelligence, may facilitate earlier identification, prompting biochemical confirmation of the diagnosis. Biochemical evaluation has benefited from advances in hormone assay harmonization and the establishment of robust age-adjusted reference ranges. Serum IGF-I is the preferred initial screening test due to its stability and reflection of integrated GH secretion. However, interpretation of assay values should consider age, sex, assay variability, and confounding conditions such as diabetes, liver or renal disease, obesity, pregnancy, and estrogen exposure. For discordant biochemical and clinical findings, it is recommended to repeat IGF-I and to measure GH during an oral glucose tolerance test (OGTT). Although random GH levels are often elevated and correlate with somatotroph adenoma size, GH suppression during OGTT is the gold-standard confirmatory test, especially in patients with borderline results. The use of ultrasensitive GH assays has lowered the recommended nadir GH cut-off threshold to ∼0.4 µg/L, with assay-specific considerations. Advances in high-resolution MRI and PET/MRI, alongside AI-driven facial recognition, electronic medical record analysis, and radiomics, offer promising avenues for earlier and more accurate diagnosis of acromegaly.

Acromegaly is a rare, chronic, and heterogeneous multisystem disorder caused by prolonged hypersecretion of growth hormone (GH) and insulin-like growth factor 1 (IGF-I), most commonly due to a pituitary somatotroph adenoma (1-3). Its clinical expression includes musculoskeletal, cardiovascular, metabolic, respiratory, dermatologic, and neuropsychiatric manifestations, reflecting the widespread physiological effects of GH and IGF-I. Although classical features such as acral enlargement and facial coarsening remain central to the diagnostic impression (Fig. 1), many patients initially present with nonspecific symptoms such as sleep apnea, carpal tunnel syndrome, arthralgias, and metabolic dysfunction. These manifestations may come to medical attention long before characteristic physical changes are recognized, contributing to the delayed diagnosis of the disease.

**Figure 1:**
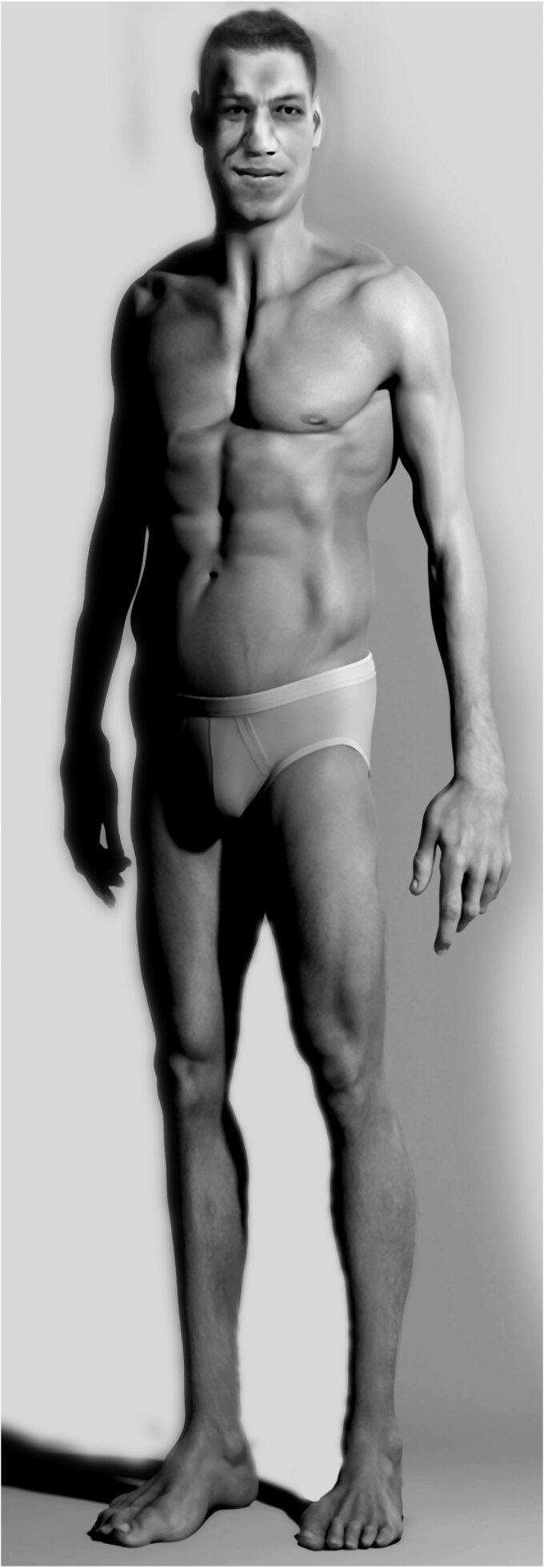
Schematic representation of the morphologic changes observed in acromegaly. Reprinted with permission from Kamenicky P, et al *Endocr Rev*. 2014;35(2):234-281 (4) with permission from Oxford University Press.

Despite progress in GH and IGF-I assay standardization, high-resolution magnetic resonance imaging (MRI), and increased awareness among endocrinologists, diagnostic delay continues to be reported in historical and contemporary cohorts. One of the main reasons for the long diagnostic delay is the rarity of the disease. Indeed, its prevalence is estimated at 5.9 out of 100 000 (5). In France, it was estimated that, statistically, a general practitioner will see only one patient during an entire career! Earlier studies described delays of 10 to 20 years, and more recent analyses demonstrate gaps of 6 to 10 years between symptom onset and diagnosis (6-12). Frequent discrepancies between patient-reported and physician-documented symptoms, as well as sex-specific presentation patterns, may contribute to this gap, and, even if the importance of snoring, weight gain, and carpal or cubital tunnel syndrome as early indicators is emphasized, their lack of specificity might explain why they are not taken into account in the diagnostic process (9, 13). It remains to be determined whether comorbidity clusters, particularly combinations of cardiometabolic, respiratory, and neurologic features, could be helpful for prompting earlier IGF-I testing even in patients who lack overt acral changes.

Concurrently, advances in diagnostic tools continue to refine early detection. Harmonized IGF-I assays and improvement in the establishment of normative values, for IGF-I as well as GH during GH suppression testing, contribute to a more reliable biochemical assessment (14-19). Imaging innovations, including high-resolution MRI and emerging positron emission tomography (PET)/MRI approaches, enhance the ability to detect subtle or atypical adenomas (20). Moreover, artificial intelligence (AI) systems, such as deep-learning facial recognition tools, electronic medical record (EMR)-based pattern-recognition algorithms (21), and radiomics-assisted image interpretation (22), offer new opportunities to identify patients with a higher likelihood proactively. The objective of this review is to integrate evidence across clinical features, comorbidity clustering, biochemical testing, imaging modalities, and AI to propose an updated framework for a timely and effective diagnosis of acromegaly.

## Clinical presentation: expanding beyond classic features

### Classic morphologic features

**Figure 2:**
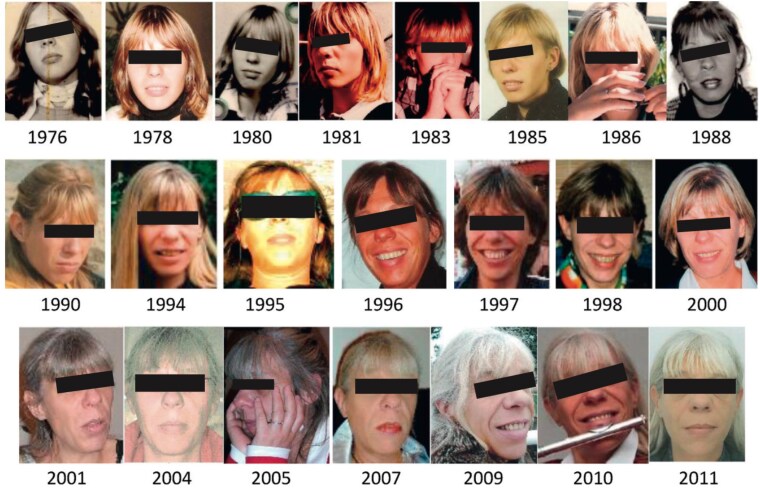
Morphologic features of a patient with acromegaly over time. Diagnosis of acromegaly was made in 2011 at the age of 50; at that time, the patient had already developed a multinodular goiter, a carpal tunnel syndrome, an adenomatous colonic polyp, and she was depressive from many years. Permission received from the patient for use of the photographs. Reprinted from Chanson P, Salenave S, Kamenicky P. Acromegaly. In: Fliers E, Korbonits M, Romijn JA, eds. *Handbook of Clinical Neurology*. Waltham, MA: Elsevier B.V.; 2014;124:197-219 (1) with permission from Elsevier.

**Figure 3:**
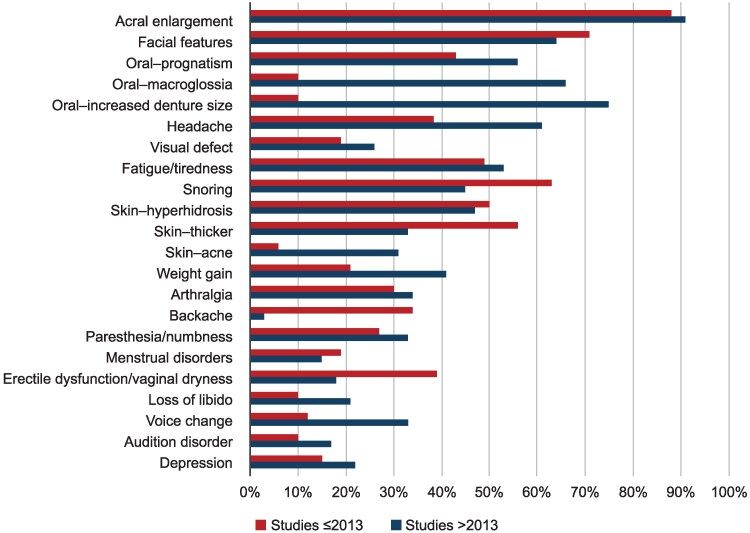
Clinical signs and symptoms at the time of diagnosis according to publication date. Note that in more recently published cohorts (after 2013), some signs and symptoms receive more attention from physicians than others, compared with more historical series published prior to 2013. Adapted from Slagboom TNA, et al *Pituitary*. 2023;26(4):319-332 (23). Under Creative Commons Attribution 4.0 License, https://creativecommons.org/licenses/by/4.0/.

Acromegaly has traditionally been recognized by its characteristic morphologic features, including acral enlargement, facial coarsening, prognathism, and macroglossia, all of which result from chronic GH and IGF-I excess. These hallmark traits remain important visual cues for clinicians; however, they often emerge late in the disease course and progress very slowly (Fig. 2), often hampering their recognition by the patient, family, or even their physician. The diagnosis is often suggested at the time of consultation with a new physician who is seeing the patient for the first time (11, 12). According to a recent meta-analysis (23), at the time of diagnosis, acral enlargement is found in 90% of patients, often leading to increased ring diameter and foot size. Facial features are characteristic in 65% of patients, including prognathism and jaw enlargement in 56% and nose enlargement in 29% (Fig. 3). Many patients do not present with overt changes during earlier stages of the disease (13). Because these features develop gradually over many years, relying solely on classic physical changes contributes to diagnostic delay (10).

### Functional symptoms and early manifestations

Functional symptoms frequently precede visible morphologic changes and may be among the earliest clues to underlying GH excess. Sleep apnea, weight gain, arthralgia, and carpal or cubital tunnel syndrome appear an average of 5 to 10 years before diagnosis (13). Headache, fatigue, and constipation also emerge early and contribute substantially to patient morbidity. Psychiatric symptoms, such as anxiety, depression, and other mood disturbances, may also represent early and under-recognized manifestations of acromegaly that can precede the diagnosis. Even if often more frequent than observed in the general population (eg, headache occurs in 59% of patients with acromegaly vs 53% in the general population, hyperhidrosis 47% vs 16%, fatigue 53% vs 35%) (23), these manifestations are common in general medical practice and may be misattributed to aging, obesity, degenerative joint disease, or lifestyle factors, which reinforces the need for heightened clinical suspicion when these features cluster together. Nevertheless, multiple correspondence analyses did not identify specific clusters of signs and symptoms (13). Screening algorithms, based on a combination of suggestive signs and symptoms, have been developed to detect acromegaly in large populations of individuals at high risk using data mining (24-26).

### Sex differences in presentation

Women more commonly present with headaches, thyroid nodules, carpal or cubital tunnel syndrome, and constipation, whereas men more often exhibit prognathism, sleep apnea, and cardiovascular manifestations such as myocardial hypertrophy or heart failure (13). The diagnosis in women is made approximately 4.5 years later than in men, with a diagnostic delay that is 2 to 4 years longer compared with male patients. These findings are consistent with prior literature suggesting sex-based variability in the GH/IGF-I levels ratio (at diagnosis, women have a higher GH relative to IGF-I level than men), symptom perception, and comorbidity burden (12, 27).

### Patient–physician symptom discrepancies

Patient-reported and physician-documented symptoms are not uniformly congruent (9, 13). Up to 36% of manifestations, particularly snoring, weight gain, fatigue, arthralgia, and loss of libido, were underreported in the medical records compared with patient questionnaires. These discrepancies likely reflect the nonspecific nature of early symptoms, patient reluctance to report certain manifestations, and clinician focus on more recognizable morphologic features. This gap highlights the value of systematic symptom elicitation and structured history-taking to capture the full spectrum of disease burden.

### Comorbidity clusters as diagnostic clues

Because acromegaly affects multiple organ systems, comorbidity clusters may serve as early diagnostic signals even in the absence of classic acral changes. Hypertension, diabetes, and dyslipidemia (23) are common and may appear years before biochemical confirmation of disease. Snoring and obstructive sleep apnea (OSA) are highly prevalent and strongly associated with early soft-tissue overgrowth (23). Carpal tunnel syndrome and peripheral neuropathies further contribute to the multisystem diagnostic pattern (23). Identifying these clusters, particularly when they occur in younger individuals or those without obesity, can prompt earlier IGF-I testing and reduce diagnostic delay. Recent machine-learning studies using EMR data reinforce the diagnostic value of comorbidity clustering by demonstrating that combinations of seemingly common conditions could reliably identify patients with a higher likelihood of acromegaly (26).

## Diagnostic delay: causes, patterns, and opportunities

Diagnostic delay is a persistent challenge in acromegaly and contributes directly to increased morbidity and mortality (7). Although diagnostic methods have improved, many patients continue to experience long intervals between the onset of symptoms and confirmation of the disease. When patient-directed questionnaires are used, the average delay is approximately 14 years from the first manifestation to diagnosis, which reflects the slow and subtle evolution of clinical features (13). Earlier cohort studies similarly described delays ranging from 6 to 20 years, depending on the clinical setting (10, 12). It is estimated to be >10 years in 24% of patients (7). Several factors contribute to this problem: rarity of the condition, unfamiliarity of physicians with acromegaly, and absence of pathognomonic symptoms at the beginning of the disease. Early onset OSA, osteoarthritis, and carpal tunnel syndrome can lead to misattribution and fragmented care across multiple specialties, without recognition of their shared hormonal etiology.

Documentation gaps (13) reduce opportunities for clinicians to identify symptom patterns that might suggest GH excess and for emerging tools based on EMR analysis and machine learning (ML) to identify high-risk clinical profiles that could support earlier detection (24-26). Continued efforts to improve symptom recognition, documentation, and cross-specialty awareness may reduce diagnostic delay and improve outcomes.

## Biochemical diagnosis: current standards

As shown in Table 1, since the first acromegaly consensus statement >25 years ago (28), criteria for biochemical diagnosis of acromegaly have changed over time (29-31). IGF-I has become more important compared with GH suppression by oral glucose tolerance test (OGTT), which in the past was the reference test for the diagnosis of acromegaly.

**Table 1:** Evolution of criteria for acromegaly diagnosis

	Year of publication	Diagnosis
1st Acromegaly Consensus Statement (28)	2000	IGF-I elevated for age and sex Confirm with random GH ≥ 0.4 μg/L *or* IGF-I elevated for age and sex Confirm with GH > 1 μg/L during OGTT
7th Acromegaly Consensus Statement (29)	2010	IGF-I elevated for age and sex *and* Random GH elevated
Endocrine Society Guidelines (30)	2014	IGF-I elevated for age Confirm with GH > 1 μg/L during OGTT
14th Acromegaly Consensus Statement (31)	2024	IGF-I > 1.3× ULN for age *and* Characteristic clinical signs of disease For equivocal results, IGF-I measurements can be repeated, *and* OGTT might additionally be useful

Abbreviations: GH, growth hormone; IGF-I, insulin-like growth factor 1; OGTT, oral glucose tolerance test; ULN, upper limit of normal.

### IGF-I as the first-line test

Serum IGF-I remains the recommended initial biochemical test for evaluating suspected acromegaly because it reflects integrated GH secretion and is relatively stable throughout the day, which allows measurement at any time, without needing to fast (14). Serum IGF-I is currently measured using the pure recombinant international standard 02/254 World Health Organization reference standard (14, 29). Normative age-adjusted data have been provided in large populations using various immunoassays (15, 16, 32, 33). The use of age-adjusted reference intervals and *Z*-scores is essential for accurate interpretation, particularly because IGF-I levels vary by age, sex, and population (34). Significant interassay and interlaboratory variability may persist, influencing diagnostic reliability (18, 35).

IGF-I measurements may also be altered by puberty, poorly controlled diabetes, liver disease, renal insufficiency, malnutrition, pregnancy, treatment with oral estrogens or selective estrogen receptor modulators, and obesity, which can lead to false-positive or false-negative results (14, 18, 19).

According to the last Acromegaly Consensus (31), the diagnosis of acromegaly is based on a serum level of IGF-I > 1.3× the upper limit of normal for age in a patient with characteristic clinical signs of the disease. Repeat measurement is recommended when IGF-I values do not align with the clinical presentation, and OGTT might also be useful.

### GH suppression testing

The introduction of international GH assay standards has minimized GH variability, and manufacturers should calibrate GH assays with the international standard 98/574 (14). The latest assays are highly sensitive with low interassay coefficients of variation.

In most patients, serum GH levels are unequivocally elevated (36) and correlate with adenoma diameter (11). However, even if increased random GH levels are generally indicative of acromegaly, normal healthy individuals may have peak GH levels in the same range, supporting the need for evaluation of GH suppression during OGTT, the gold-standard confirmatory test (37), particularly in patients with modestly elevated or borderline IGF-I levels. Advances in ultrasensitive GH assays have resulted in lower and more precise GH nadir thresholds, improving diagnostic accuracy (18). Rather than the 1-µg/L threshold recommended by the Endocrine Society clinical practice guideline >10 years ago (30), a nadir serum GH level less than 0.4 µg/L should be used as a normal response to glucose load when an ultrasensitive assay is used (38, 39). Moreover, this threshold may be further refined according to assay kit (40), sex, body mass index, and the use of estro-progestative oral medications (17) when defining the threshold for GH under OGTT. Obesity, advanced age, and uncontrolled diabetes mellitus may blunt GH suppression and make results more difficult to interpret. The last Acromegaly Consensus (31) now incorporates assay-specific GH nadir cutoffs and emphasizes the need for harmonization across laboratories.

About one-third of patients with acromegaly display a paradoxical increase of GH under OGTT (37), linked to somatotroph adenoma expression of glucose-dependent insulinotropic polypeptide receptor (41), by analogy to glucose-dependent insulinotropic polypeptide–dependent Cushing's syndrome (42). Their IGF-I levels are higher, and their adenoma is smaller and more often densely granulated (43).

Very rarely, patients, generally with microadenomas and/or at the onset of the disease, who have functional symptoms and moderate clinical signs of acromegaly, show high IGF-I levels but low GH levels that can suppress to <0.4 µg/L during the OGTT (44).

### Additional biochemical markers

Additional biochemical markers may provide adjunctive information in selected clinical settings. Prolactin elevation is common in mixed somatotroph lactotroph adenomas and may aid in adenoma characterization and treatment planning. There is no benefit to measure IGF-binding protein 3 and to perform GH profile testing, except in patients with diabetes mellitus for whom OGTT is not recommended (45). Soluble alpha Klotho, a biomarker of GH action, also may reflect disease activity (46, 47).

### Biochemical diagnosis in special populations

Biochemical interpretation requires special consideration in populations with altered GH–IGF-I axis physiology. Older adults exhibit lower IGF-I and GH levels, whereas individuals with obesity or insulin resistance may have suppressed GH despite active acromegaly. In patients with uncontrolled diabetes mellitus, serum IGF-I may be falsely normal or low (48). It is thus recommended, if the clinical suspicion of acromegaly is high, to verify whether IGF-I levels increase after achieving control of diabetes mellitus, using insulin if necessary. During pregnancy, placental GH production and physiological changes in IGF-I metabolism complicate the interpretation of IGF-I and GH suppression tests. Patients with chronic kidney disease or severe liver dysfunction may have abnormal IGF-I clearance or synthesis (18, 19). In these situations, clinicians often rely on a combination of repeated biochemical testing and imaging findings to reach a definitive diagnosis (31).

### Acromegaly phenotype with normal GH–IGF-I axis

Some individuals present with clinical acromegaly features but normal IGF-I levels and suppressible GH concentrations under OGTT. This may be caused by spontaneously resolving true acromegaly, after apoplexy of a prior somatotroph adenoma, or be acromegaloid features observed with severe insulin resistance, profound hypothyroidism, congenital generalized lipodystrophy (49), or rare overgrowth pachydermoperiostosis (50-52).

## Imaging advances in acromegaly

Diagnosis and management of pituitary adenomas are benefiting greatly from the technical progress of MRI, functional PET, and radiomics (53, 54).

### MRI pituitary: standard approaches

Contrast-enhanced MRI of the pituitary gland is the standard structural imaging modality for evaluating suspected somatotroph adenomas. High-resolution T1-weighted sequences with gadolinium enhancement allow detailed sella visualization (54-56). Microadenomas (≤10-mm diameter) typically present as small hypoenhancing lesions relative to normal pituitary tissue, whereas macroadenomas (>10 mm) are more likely to demonstrate suprasellar extension, cavernous sinus involvement, or compression of the optic chiasm. These characteristics assist in establishing the diagnosis, determining surgical feasibility, and assessing the risk of mass effect.

About 70% of patients with acromegaly have a pituitary macroadenoma (11), which is often invasive, especially in the cavernous sinus (57). Macroadenomas usually extend inferiorly in the vertical plane as compared with non-functioning pituitary adenomas (57, 58). Importantly, imaging often underestimates true adenomatous invasion, which will be re-assessed at surgery and histopathology, and ultimately, even if the surgeon claims to have performed a total gross removal of the lesion, at hormonal postoperative evaluation.

Somatotroph adenomas may be hypointense, isointense, or hyperintense on T2-weighted MRI sequences (57). Hypointense imaging on T2-weighted MRI may predict a favorable outcome after somatostatin receptor ligand treatment (59-61). However, this was not confirmed by a recent prospective study (62).

When MRI is contraindicated or the patient is claustrophobic, skull base computed tomography may be used. If necessary, this technique may demonstrate the size and extension of the pituitary mass for presurgical evaluation.

### High-resolution and dynamic MRI techniques

Advances in MRI technology have improved diagnostic sensitivity. Superior spatial and contrast resolution are provided with 3-Tesla (3T) MRI compared with 1.5T MRI, which enhances detection of small microadenomas that may not be visible on lower-field systems (54-56). Dynamic contrast-enhanced MRI, performed during early contrast transit, further improves identification of subtle lesions due to differential enhancement patterns between adenomatous and normal pituitary tissue. Volumetric imaging and multiplanar reconstructions also contribute to improved visualization of adenoma margins and parasellar extension. These techniques are particularly valuable in patients with biochemical evidence of acromegaly but nondiagnostic findings on routine MRI. In particular, 7T MRI could be helpful to improve preoperative determination of cavernous sinus invasion (63).

### Functional imaging

Functional imaging is an important adjunct when structural MRI results are inconclusive (54). PET with ^11^C-methionine is highly sensitive for detecting metabolically active pituitary adenomas, especially in patients with “MRI-negative” acromegaly, because adenomatous tissue exhibits increased amino acid transport and protein synthesis (64, 65), but the short tracer half-life limits availability of the technique as it requires an on-site cyclotron. ^18^F-fluoroethyltyrosine PET/MRI is more readily available and is promising for detecting pituitary adenomas (66, 67).

## How to improve acromegaly screening: use of comorbidity and symptom clusters

Recognition of acromegaly based solely on acral enlargement or facial coarsening is often insufficient because these morphologic changes typically are detectable late in the disease course. In this setting, screening for this disease in patients with comorbidities and/or signs or symptoms known to be associated with acromegaly could be relevant.

### Acromegaly screening in patients with a single comorbidity known to be associated with GH excess

Screening for acromegaly in patient populations with comorbidities known to be associated with acromegaly, including diabetes mellitus, sleep apnea, or acral enlargement, has not been useful. In 2270 Brazilian patients with type 2 diabetes mellitus or impaired glucose tolerance, 56 had elevated IGF-I levels. Of those patients, 6 had unsuppressed GH levels during OGTT, and 2 had an abnormal MRI, giving an estimated acromegaly prevalence of 1% (68). In a Japanese population of 327 hospitalized patients with type 2 diabetes mellitus, the prevalence of acromegaly was 0.6% (69). In 178 of 17 700 primary care patients who responded to a questionnaire evaluating change in ring or foot size in the previous 5 years, IGF-I was increased in 8 and acromegaly confirmed in 6, with a prevalence of 3% (70). In 873 patients referred to sleep clinics in France, 8 had increased IGF-I levels, of whom 4 had non-suppressed GH levels under OGTT, and 2 had an abnormal MRI, resulting in a prevalence of 0.25% and 0.35% when sleep apnea was suspected or confirmed, respectively (71). In 507 German patients with sleep apnea, there was a 0.2% acromegaly prevalence (72). In 6773 primary care patients, IGF-I was increased in 125 and acromegaly confirmed in 7 (prevalence of 0.1%) (73).

Even if the risk of presenting with acromegaly is greatly increased in patients with diabetes, sleep apnea syndrome, or acral enlargement compared with the general population, considering the very low prevalence of acromegaly in these populations, screening for GH excess by measuring IGF-I in these populations has little utility (74). Moreover, to adopt large-scale disease screening, the World Health Organization requires rigorous criteria to be met (75), which is challenging for acromegaly.

### Could clusters of symptoms or comorbidities be helpful?

Large cohort studies demonstrate that clusters of common comorbidities and symptoms could serve as early diagnostic signals even when classic phenotypic features are not yet evident (13, 23, 76). Early manifestations frequently include sleep-disordered breathing, musculoskeletal complaints, metabolic abnormalities, and neurologic syndromes that accumulate gradually over time and often prompt evaluations by multiple specialists before the unifying diagnosis is considered (77).

Snoring, OSA, and fatigue frequently occur in conjunction with hyperhidrosis, forming a cluster commonly observed in early acromegaly. Neuroskeletal clusters, including carpal tunnel syndrome, arthralgia, and recurrent headaches, may reflect early soft-tissue hypertrophy and joint involvement and often predate morphologic changes. Cardiometabolic clusters such as type 2 diabetes, hypertension, and sleep apnea are highly prevalent among patients ultimately diagnosed with acromegaly and frequently lead to fragmented evaluations across primary care, cardiology, and sleep medicine. In women, clusters that include weight gain, snoring, menstrual disturbances, and thyroid nodules have been reported more frequently and could heighten suspicion even in the absence of overt acral enlargement (13, 23, 76). Thus, evaluating combinations of common symptoms rather than isolated findings should enable earlier recognition.

### Screening tools using clusters

Several structured tools leverage symptom and comorbidity clusters to improve early detection. The ACROSCORE screening algorithm incorporates sleep-disordered breathing, joint symptoms, acral changes, and metabolic abnormalities into a quantitative score that has demonstrated good diagnostic performance in outpatient settings (78). However, the suspicion of acromegaly is what leads to the use of ACROSCORE, and when acromegaly is suspected, IGF-I is the optimal diagnostic screening test. In the ACROTEST study, where multiple correspondence analyses evaluated associations of signs and symptoms at diagnosis, no specific cluster was identified (13). Most physicians do not reliably detect acromegaly due to the rarity of the disease and the small number of unique pathognomonic signs, contrasting with many nonspecific signs and symptoms. In this context, data mining of large population sets could offer integrated comorbidity clusters into EMR-based algorithms to improve case identification and support earlier biochemical testing (26, 79).

## Artificial intelligence and machine learning for earlier detection

### Facial recognition algorithms

AI-based facial analysis has emerged as a promising approach for early detection of acromegaly. Convolutional neural networks trained on annotated facial images have demonstrated high diagnostic accuracy in distinguishing individuals with subtle acromegaly features from healthy controls (80-85). These models may detect disease-related craniofacial changes years (mean, 7.47 years) before clinical recognition, suggesting that facial analytics could support earlier detection in populations undergoing routine imaging or biometric screening (86). More advanced deep-learning systems, such as the AcroFace platform, incorporate next-generation architectures capable of identifying changes across multiple facial regions, even in patients without classic acral enlargement (87). These systems require careful evaluation of demographic biases, privacy concerns, and ethical implications related to automated facial analysis. The necessity for robust multi-ethnic training datasets and transparent interpretability remains an important focus of ongoing research (88).

### Voice analysis

A peculiarity of patients with acromegaly is the very specific voice changes (hoarse deep voice related to lower fundamental frequency of vocal fold cycles and higher perturbation markers than individuals without acromegaly) that can be captured by voice software (89). In voice recordings of Swedish patients and controls studied using broad acoustic analysis and machine learning, the machine learning model identified patients with acromegaly with higher accuracy than experienced endocrinologists (90).

### EMR-embedded algorithms

ML models applied to EMR data offer an avenue for early detection. These systems leverage large volumes of structured information, including *International Classification of Diseases* codes, laboratory values, vital-sign trajectories, pharmacy data, and consultation patterns. In a large Sicilian database, EMR-based ML algorithms proved to have highly consistent diagnostic accuracy (24). Conditional and unconditional penalized multivariable logistic regression models and 3 ML algorithms for identifying combinations of potential predictors of acromegaly diagnosis were derived from claims databases (25). Overall, only the immunosuppressant-related pharmacy claims were selected as a diagnosis predictor by all 5 models and algorithms, suggesting that systemic inflammation (eg, in the context of osteoarthropathy or misinterpretation of other signs and symptoms of acromegaly) may predict diagnosis of the disease. Other predictors selected by ≥2 models were combined to develop a meta-score with a moderate discrimination accuracy (25). In another study, Dutch investigators extracted data from the EMR of a tertiary hospital center and predictors derived from literature review, real-world evidence, and expert opinion were included in the algorithm. The best algorithm enabled the detection of 50% of patients with previously diagnosed acromegaly and identified 0.1% of the hospital population as potentially having acromegaly. Given that this figure is 10 times higher than the estimated prevalence of acromegaly, further improvement and validation of this algorithm are warranted (26). Such approaches could enable automated flags in clinical workflows, prompting IGF-I measurement for patients who present with multisystemic symptom clusters even in the absence of typical phenotypic features. These tools, even if very attractive, clearly necessitate refinement.

### Biochemical pattern recognition

AI and ML approaches have been applied to the interpretation of complex biochemical datasets, particularly for predicting response to therapy (21, 91). Predictive models incorporating IGF-I values, IGF-I Z scores, GH suppression dynamics, age, obesity, glucose tolerance, and liver or renal function could potentially estimate the probability of acromegaly with borderline or discordant biochemical results.

### Radiomics and imaging-based AI

Radiomics techniques apply ML algorithms to extract quantitative imaging features not visible to the human eye. Signatures derived from dynamic contrast-enhanced MRI may differentiate microadenomas from normal gland tissue with high accuracy, even in patients previously labeled as “MRI negative” (20-22, 54). These signatures include texture heterogeneity, shape descriptors, contrast kinetics, and voxel-based intensity patterns and could also be combined with PET-based ^11^C-methionine or ^18^F-fluoroethyltyrosine uptake patterns. Deep-learning models integrating volumetric pituitary imaging are being explored for fully automated localization and segmentation (20-22, 54). Imaging interpretation combining human expertise with algorithmic pattern recognition should improve diagnostic sensitivity.

### Future integration

The future of AI-enabled detection in acromegaly lies in multimodal platform integration to provide real-time clinical decision support. Diagnostic frameworks could combine facial and voice analytics, EMR-derived comorbidity clustering, biochemical risk modeling, and radiomic imaging signatures to create unified predictive models (88). When implemented responsibly, these technologies have the potential to reduce diagnostic delay, improve the accuracy of biochemical and imaging interpretation, and support earlier referral to endocrinologists. Large-scale validation studies, attention to algorithmic equity, and incorporation into clinical workflows will be essential for translating these tools into routine practice (92, 93).

## How to improve acromegaly screening by non-endocrinologists

Acromegaly frequently manifests in specialty settings outside of endocrinology, making coordinated cross-disciplinary screening essential. Sleep medicine specialists should consider screening patients with moderate to severe OSA, particularly when accompanied by craniofacial changes or metabolic abnormalities (74).

In rheumatology or radiology, warning signs include bilateral or recurrent carpal tunnel syndrome, early-onset osteoarthritis, and disproportionate arthropathy or unusual arthropathy sites (eg, shoulder)—patterns repeatedly described in acromegaly cohorts (94).

Dentistry is positioned to detect early craniofacial manifestations such as mandibular prognathism, interdental spacing, malocclusion, and macroglossia; endocrine referral is warranted when ≥1 of these findings arises in a patient (95, 96).

In cardiology, screening should be considered in adults presenting with unexplained concentric left ventricular hypertrophy or diastolic dysfunction, hypertension, or arrhythmias, given the strong association between GH/IGF-I excess and cardiovascular morbidity (23). However, cardiomyopathy in acromegaly has become less prevalent (97). The declining prevalence of cardiomyopathy in acromegaly is attributed to earlier diagnosis, changes in imaging methodologies over time, improved biochemical control of GH and IGF-I excess, and advances in medical and surgical management that prevent long-term cardiac remodeling.

Primary care providers should combine visual assessment, symptom clusters, and low-threshold IGF-I testing to capture early or otherwise unrecognized patients. However, the rarity of the disease makes it highly unlikely that physicians have ever encountered such a patient (3), characteristic of the challenge of screening for rare diseases in general and acromegaly in particular. AI-based EMR alerts may enhance early recognition, provided that relevant data have been entered into the systems before data mining.

## Special situations

### MRI-negative acromegaly

Some patients with biochemically confirmed acromegaly have no discrete adenoma visible on standard pituitary MRI. Microadenomas of <2 to 3 mm often escape detection even with conventional 1.5T imaging, requiring dynamic contrast-enhanced and 3T MRI for improved sensitivity. Radiomic models and advanced image post-processing may further enhance visualization of subtle lesions (53, 54). When MRI remains negative, pituitary origin should be distinguished from ectopic GH releasing-hormone (GHRH)–mediated GH excess (see below); ectopic somatotroph adenomas in the sphenoid sinus, clivus, cavernous sinuses, suprasellar region, or nasopharynx (98); or, even more rarely, GH-secreting ectopic tumors such as pancreatic (99), bronchial (100, 101), and ovarian tumors (102); or non-Hodgkin's lymphoma (103).

### Ectopic GHRH secretion

Diffuse pituitary enlargement without a visible focal adenoma should raise suspicion for somatotroph hyperplasia due to ectopic GHRH secretion, which accounts for <1% of acromegaly cases (2, 3, 104). Biochemical clues include high plasma GHRH and evidence of pituitary hyperplasia. Identification of a GHRH-secreting neuroendocrine tumor (NET) requires targeted chest and abdominal imaging (104) and may be enhanced by ^68^Ga-DOTATATE PET, which has high sensitivity for somatostatin-avid NETs.

The diagnosis is challenging in the setting of multiple endocrine neoplasia type 1 (MEN1) in which a pituitary somatotroph adenoma is suspected to be a component of the typical MEN1 triad (ie, hyperparathyroidism, pituitary adenoma, and pancreatic NET) (105), while in fact the acromegaly is related to ectopic GHRH secretion by a pancreatic or bronchial NET (104).

### Pediatric and young adult presentations

Acromegaly presenting in childhood or adolescence is rare and clinically distinct, manifesting as gigantism when occurring before epiphyseal closure. These patients show more aggressive adenoma behavior, higher GH levels, and earlier clinical progression (106-109). IGF-I interpretation in this age group requires strict age- and puberty-adjusted normative ranges, given physiological adolescent GH surges (18, 19). Children carry a higher genetic predisposition, such as *AIP* mutations (106-110).

### Syndromic settings (MEN1 and MEN4, McCune-Albright syndrome, Carney complex)

Acromegaly may arise in patients with hereditary endocrine tumor syndromes. In MEN1, GH-secreting adenomas occur with parathyroid hyperplasia and pancreatic NETs, often presenting at a younger age (105, 111, 112). Acromegaly may also be part of autosomal dominant MEN4, caused by mutations in *CDKN1B* and associated with primary hyperparathyroidism (113) or with familial paraganglioma or pheochromocytoma (“MEN5”) (114). McCune-Albright syndrome, characterized by fibrous bone dysplasia, cafe-au-lait skin tags, and precocious puberty, may also feature GH excess due to somatotroph hyperplasia and/or adenoma (115). In Carney complex, a very rare syndrome linked to inactivating heterozygous *PRKAR1A* mutations, acromegaly is associated with primary pigmented adrenal nodules with Cushing's syndrome; thyroid, cardiac, cutaneous, and mucosal myxomas; and pigmented cutaneous lesions (116).

## Conclusion

Criteria for diagnosing acromegaly are well-defined, and the diagnosis is relatively straightforward once one thinks about it. The challenge for the physician is to be aware of the diagnosis, because early signs and symptoms are not specific at the onset of the disease and are thus largely overlooked. Because the disorder is so uncommon, even when the clinical picture is characteristic, it is rarely recognized by non-endocrinologists. Acromegaly screening attempts in specific patient populations with a comorbidity associated with acromegaly, such as diabetes, sleep apnea, or acral enlargement, have not yielded actionable results with a very low cost-effective balance. Accordingly, modern strategies should integrate comorbidity clusters, patient-reported symptoms, and emerging computational tools to capture the condition earlier and more accurately. The field is moving away from a paradigm in which diagnosis depends on late, overt appearance changes toward one grounded in the recognition of reproducible clinical, biochemical, and digital patterns that precede visible physical manifestations. By combining structured assessment of multisystem comorbidities with high-quality patient-reported information and advanced EMR-based screening, facial analysis, and radiomic features, clinicians could, hopefully, better identify individuals with acromegaly earlier in the course of their disease, thereby reducing long diagnostic delays. This pattern-based framework represents a significant shift in acromegaly detection and holds promise for improving timely, patient-centered care.

## Data Availability

Data sharing is not applicable to this article as no data sets were generated or analyzed during the present study.

## Abbreviations

AI: artificial intelligence
EMR: electronic medical record
GH: growth hormone
GHRH: growth hormone releasing-hormone
IGF-I: insulin-like growth factor 1
MEN1: multiple endocrine neoplasia type 1
ML: machine learning
MRI: magnetic resonance imaging
NET: neuroendocrine tumor
OGTT: oral glucose tolerance test
OSA: obstructive sleep apnea
PET: positron emission tomography
T: Tesla
ULN: upper limit of normal
